# Maternal and pregnancy predictive risk factors for having a compensated maternal injury claim: a Swedish nationwide cohort study

**DOI:** 10.1038/s41598-023-49234-7

**Published:** 2023-12-08

**Authors:** A. Ivert, N. Holowko, X. Liu, M. Edqvist, N. Roos, P. Gustafson, O. Stephansson

**Affiliations:** 1 Clinical Epidemiology Division, Department of Medicine, Solna, Karolinska Institutet, K2 Medicin, Solna, K2 KEP Stephansson, Solna, 171 77 Stockholm, Sweden; 2 Department of Obstetrics and Gynecology, Karolinska University Hospital, Stockholm, Sweden; 3 Department of Clinical Sciences, Faculty of Medicine, Lund University, Lund, Sweden; 4 Löf – the National Swedish Patient Insurance Company, Stockholm, Sweden

**Keywords:** Outcomes research, Epidemiology, Disease prevention

## Abstract

To describe trends and identify maternal and pregnancy predictive risk factors for having a compensated claim for a maternal injury during delivery, as a proxy for having received suboptimal care. This nationwide retrospective cohort study included 1 754 869 births in Sweden between 2000 and 2016, including 4488 maternal injury claims filed with The National Swedish Patient Insurance Company (Löf), of which 1637 were compensated. Descriptive statistics on maternal and pregnancy characteristics, trends in filed/compensated claims over time, and distribution of compensated claims by clinical classification are presented. Characteristics associated with suboptimal care were identified using multivariable logistic regression, with mutual adjustment in the final model. Compensated claims were sorted into 14 clinical classifications (ICD-10 codes for main condition, injury, and causality). Overall, there was a two-fold increase in filed claims from 2000 to 2016, peaking in 2014. The rate of compensated claims only increased marginally, and 36.5% of filed claims were deemed avoidable. Perineal and pelvic floor injuries, as well as medical and diagnostic errors, were responsible for the majority of compensated claims. Women with a previous caesarean section, post term delivery, chronic or gestational disease, > 13 antenatal visits, or a multiple pregnancy had increased risk of having a compensated claim for a maternal injury during delivery. Understanding the risk factors for having a compensated maternal injury claim may guide health workers and maternity wards in improving the quality and organisation of care to reduce the risk of childbirth related injuries.

## Introduction

Globally, avoidable injuries in healthcare represent a significant cause for mortality and morbidity^1^. A recent systematic review and meta-analysis, including mostly studies from high-income countries (47% from the U.S. and 39% from Europe), estimated avoidable injuries in 6% of patients^2^; while within obstetrics and gynecology, this occurrence has been estimated to be as high as 50–74%^3–5^.

Avoidable injuries arise from errors in healthcare management and can result in temporary or permanent disability and extended hospital stays^6,7^. Labour itself can bring risks for both the mother and infant^8,9^, therefore quality of care and safety during childbirth is crucial for reducing morbidity and mortality among mothers and their infants^10–13^. While maternal mortality is a rare event in high-income countries^13,14^, birth complications do arise and may be due to an avoidable injury (and having received suboptimal care), the prevalence of which may change overtime. In Sweden—as evaluated by the National Patient Insurance Company (Löf)—patients who receive suboptimal health care and subsequently suffer an avoidable injury are entitled to compensation under the Swedish Patient Injury Act.

Complications during pregnancy and childbirth vary by maternal characteristics, such as advanced maternal age, chronic disease, smoking, obesity, education level, and country of birth^14–17^. However, there is a lack of knowledge on the association between maternal and pregnancy characteristics, structural factors, and the quality of the maternity care provided on the risk of avoidable injuries.

Using information on births in Sweden between 2000 and 2016 linked with insurance data for filed and compensated maternal injury claims, the objective of this study was to: (i) describe trends in the number of maternal injury claims and compensation over time; (ii) describe the types of maternal injuries compensated; and (iii) investigate maternal and pregnancy characteristics (upon admission to the delivery ward) associated with having a compensated maternal injury during childbirth.

## Methods

### Data source and study population

We used data from the national Swedish Medical Birth Register (MBR), including all births in Sweden^18^. Using the mother’s unique personal identification number (ID) and year of the index birth, the MBR was linked with data on: (i) maternal injury claims filed with Löf, and (ii) years of formal education (from the LISA database held at Statistics Sweden)^19^. Information on all filed claims for maternal injuries were identified by Löf, with the possibility of multiple claims for the same birth.

The nationwide MBR contains prospectively collected data from antenatal, delivery, and neonatal care, with most variables assessed to be of high quality^18^. Each filed injury claim was reviewed by medical advisors at Löf—experienced specialists in obstetrics/gynaecology for maternal injuries, and paediatricians for infant injuries—and included investigating patient medical records from healthcare providers. Claims are rejected when the injury is not deemed to be avoidable (according to the Swedish Patient Injury Act) or is reported more than 10 years after the injury (https://lof.se). Compensation administered by Löf is to account for patient suffering or financial losses caused by suboptimal care, with approved claims classified according to the following injury severity: sick leave < 3 months, sick leave ≥ 3 months, disability 1–15%, disability 16–30%, disability > 30%, or death.

We linked data for all births in Sweden between 2000 and 2016 with the insurance data for filed and compensated maternal injury claims. Our study population was drawn from the 1 782 802 births within our study period, with a total of 6608 claims filed for any injury during childbirth. After excluding claims reported more than 10 years after the injury, with missing maternal ID, and duplicate records, the final study population included 1 754 869 births, with 4488 filed maternal injury claims (of which 1637 were compensated and 2851 rejected) (Supplementary Figure S1). Using linked register data, this study was approved by the ethical review board in Stockholm (#2017-1308-31, granted 2017-08-16) who waived the need for written consent.

### Outcome – compensated maternal injury claim

**Figure 1:**
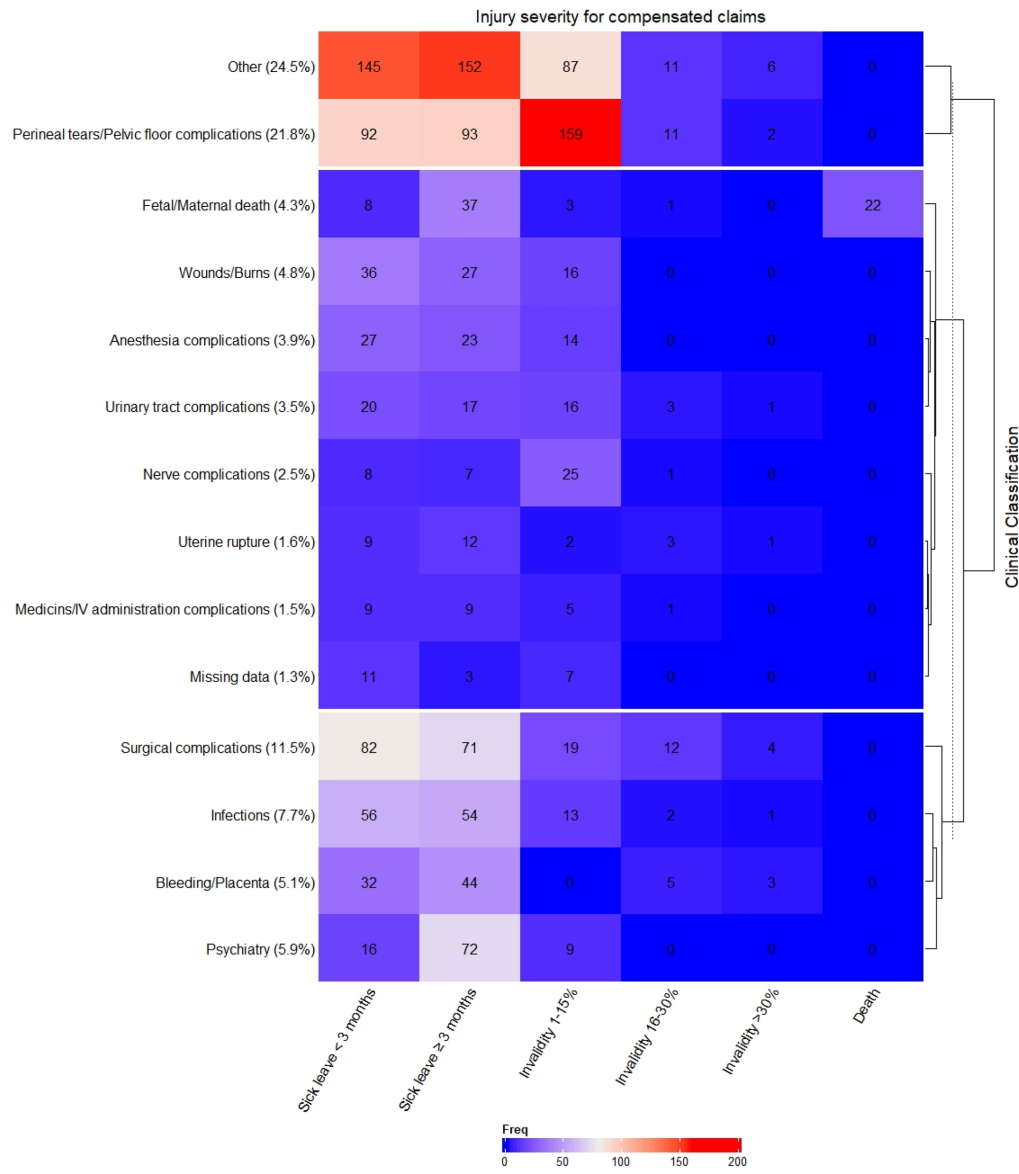
The frequency of compensated claims by injury severity (columns) and clinical classification (rows), presented in a hierarchically clustered heatmap plot (N = 1,637). Clinical classification is defined using the ICD-10 codes for the main condition, injury, and cause. The proportions of each classification group are presented, with more detailed information on the group of ‘Other’ injuries (25.7%) found in Supplementary Table S1.

Having had a compensated maternal injury claim validated by Löf was used as a proxy for suboptimal care during childbirth, given that only those injuries assessed to be avoidable were compensated. It was therefore assumed that women without a compensated claim received optimal care. To better understand the drivers of possible suboptimal care, we classified all compensated claims into 14 clinical classifications and degree of severity (including a separate category for missing) (Fig. 1), using ICD-10 codes for main condition, injury, and causality. Details of the heterogeneous category of ‘other’ injuries is included in Supplementary Table S1.

### Exposures—Maternal and pregnancy characteristics (up until admission to the delivery ward)

The following maternal and pregnancy characteristics (up until admission to the delivery ward) were extracted from the MBR and regarded as potential risk factors: maternal age, parity, previous caesarean section, maternal height, early pregnancy body mass index (BMI), smoking status at first antenatal visit, years of formal education, living with partner, region of birth (mother), chronic disease (defined as any of the following: epilepsy, asthma, kidney disease, systemic lupus erythematosus, inflammatory bowel disease (ulcerative colitis/Crohn’s disease), chronic hypertension, or diabetes), gestational disease (defined as any of the following: preeclampsia, gestational hypertension, gestational diabetes, or intrahepatic cholestasis of pregnancy), number of antenatal care visits, in vitro fertilisation, gestational age, multiple births, and calendar year. For all regression models, we combined parity and previous caesarean section into a single categorical variable: nulliparous, parous without previous caesarean section, or parous with previous caesarean section.

### Statistical analysis

**Figure 2:**
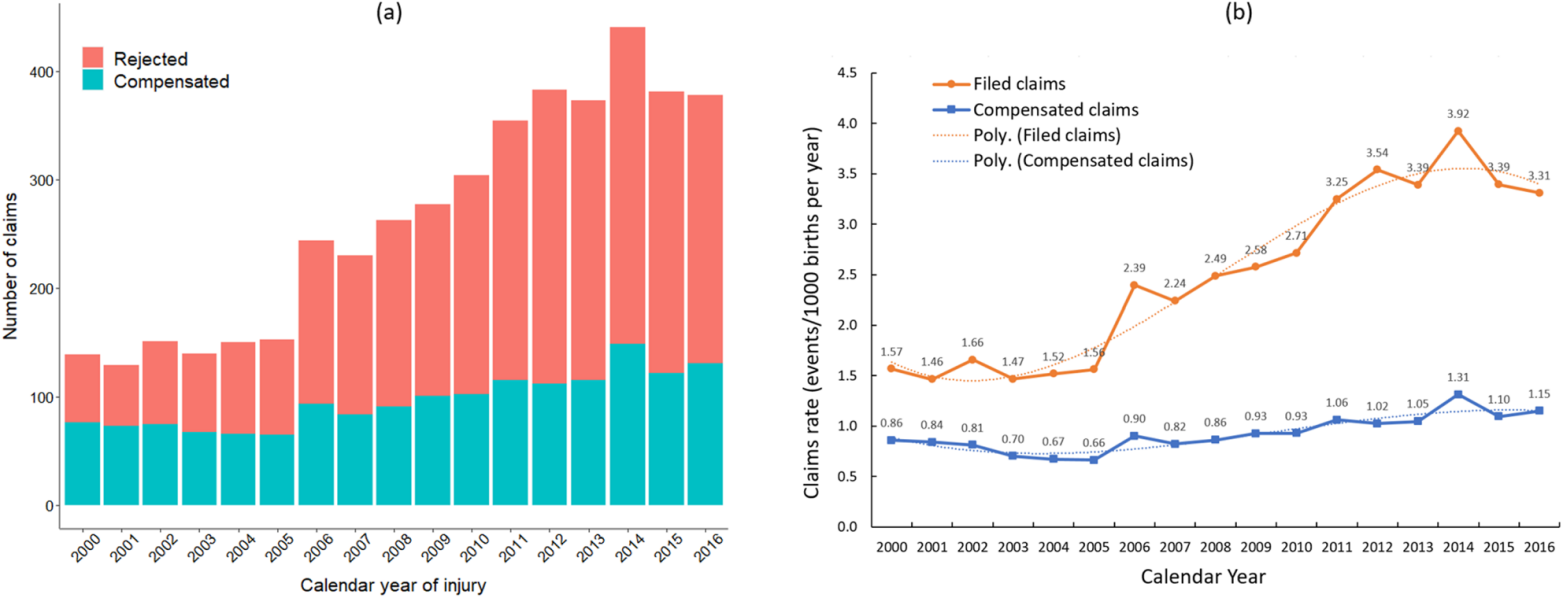
(**a**) The number of compensated and rejected maternal injury claims per calendar year from 2000 to 2016; and (**b**) Time trends of claim rates for maternal injuries from 2000 to 2016 (including polynomial trend-lines) among women giving birth in Sweden between 2000 and 2016 (N = 1 754 869).

**Figure 3:**
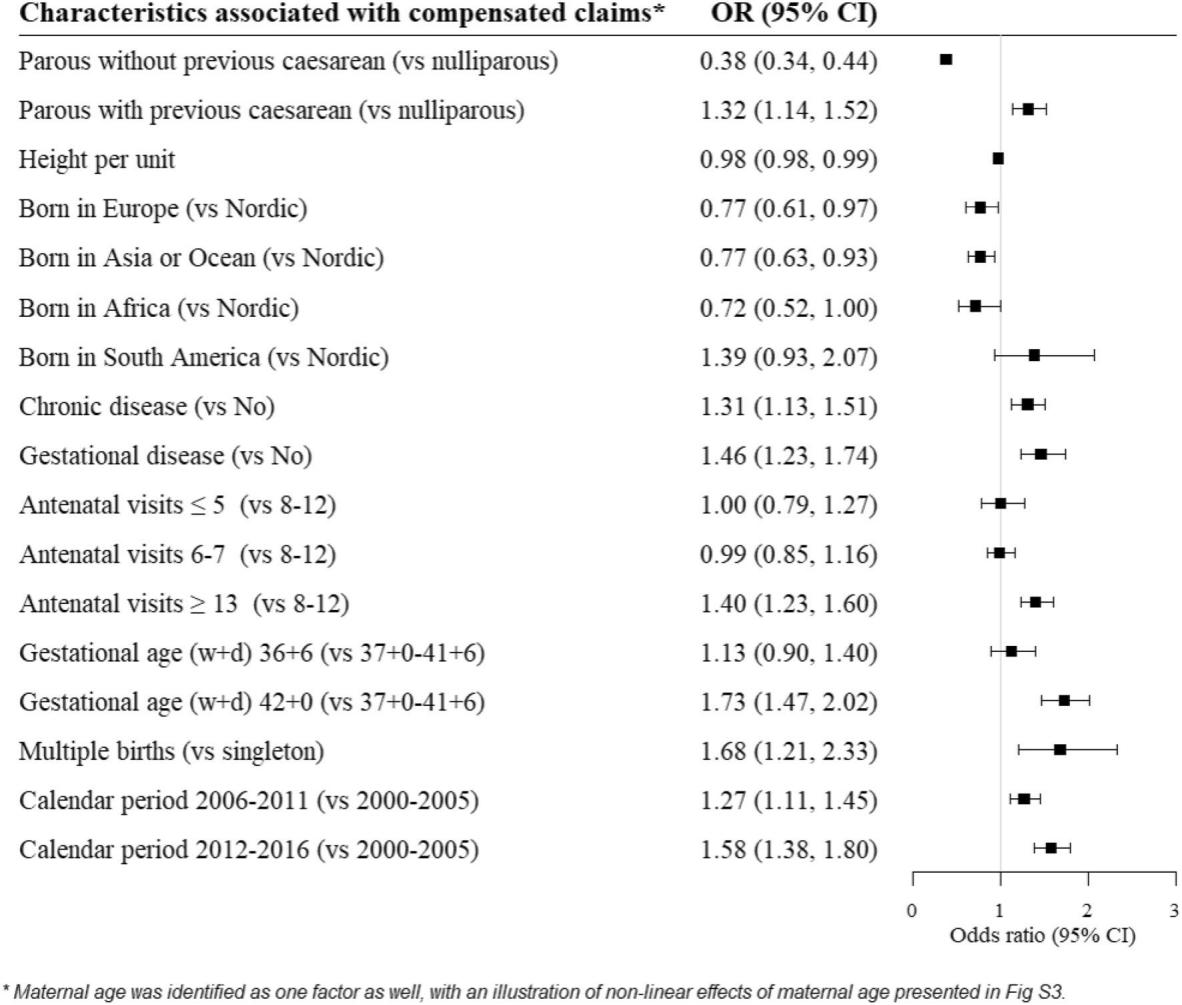
Maternal and pregnancy characteristics associated with having a compensated maternal injury claim. Starting from a full model, including all characteristics presented in Supplementary Table S2, a top-down approach using the Akaike Information Criteria was used to find the most parsimonious model, resulting in this final set of variables. The odds ratios (ORs and 95% CI) presented are estimated from the final model including only these identified variables (from a predictive standpoint).

Descriptive statistics on maternal and pregnancy characteristics are summarised in Table 1, and the number and trend of maternal injury claims throughout the study period is presented in Fig. 2. Characteristics associated with having a compensated claim were identified using multivariable logistic regression (Supplementary Table S2), with univariate estimates presented for comparison. Starting with a full model including all characteristics shown in Supplementary Table S2, a top-down approach was used to find the most parsimonious model by minimizing the Akaike Information Criteria^20^. Odds ratios (ORs) and 95% confidence intervals (CIs) are presented from the final model (Fig. 3), including only the core variables identified as being associated with having a compensated claim (including a linear term for height and quadratic term for age) (Supplementary Figure S2). Subsequently, we checked for multi-collinearity among all selected variables and investigated the variability of identified variables using a bootstrap procedure.

**Table 1:** The number of compensated and rejected maternal injury claims by maternal and pregnancy characteristics, among women giving birth in Sweden between 2000 and 2016 (= 1 754 869).

Characteristics	Mother-infant dyads ^a^ N (Column %)	Maternal injury claims	
		Compensated N (% within stratum)	Rejected N (% within stratum)
Total	1,754,869	1637	2851
**Maternal age (years) median (IQR)**		31 (27–34)	30 (26–34)
≤ 24	248,608 (14.2)	204 (0.08)	476 (0.19)
25–29	531,421 (30.3)	473 (0.09)	903 (0.17)
30–34	607,088 (34.6)	574 (0.09)	908 (0.15)
≥ 35	367,751 (21.0)	386 (0.10)	564 (0.15)
Missing	1	0	0
**Parity**			
Nulliparous (0)	776,982(44.3)	960 (0.12)	1814 (0.23)
Multiparous (≥ 1)	977,887 (55.7)	677 (0.07)	1037 (0.11)
**Maternal height (cm) median (IQR)**		165 (162–170)	166 (161–170)
≤ 159	223,066 (13.4)	214 (0.10)	424 (0.19)
160–164	422,207 (25.4)	439 (0.10)	691 (0.16)
165–169	483,289 (29.1)	444 (0.09)	781 (0.16)
≥ 170	532,860 (32.1)	444 (0.08)	804 (0.15)
Missing	93,447	96	151
**Maternal BMI** (WHO categories, kg/m^2^) median (IQR)		23.9 (21.7–27.1)	24.2 (21.7–27.6)
Underweight (< 18.5)	38,395 (2.4)	37 (0.10)	68 (0.18)
Healthy weight (18.5–24.99)	964,755 (60.6)	870 (0.09)	1453 (0.15)
Overweight (25.0–29.99)	397,210 (24.9)	379 (0.10)	696 (0.18)
Obese (≥ 30.0)	192,426 (12.1)	202 (0.10)	392 (0.20)
Missing	162,083	149	242
**Smoking status at first antenatal visit**			
Smoker	123,110 (7.4)	112 (0.09)	217 (0.18)
Non-smoker	1,547,920 (92.6)	1441 (0.09)	2497 (0.16)
Missing	83,839	84	137
**Years of formal education**			
≤ 9	191,553 (11.2)	149 (0.08)	275 (0.14)
10–12	697,500 (40.7)	630 (0.09)	1195 (0.17)
≥ 13	825,907 (48.2)	828 (0.10)	1340 (0.16)
Missing	39,909	30	41
**Living with partner**			
Yes	1,568,311 (94.0)	1452 (0.09)	2508 (0.16)
No	99,842 (6.0)	91 (0.09)	188 (0.19)
Missing	86,716	94	155
**Region of birth (mother)**			
Nordic	1,390,242 (79.4)	1342 (0.10)	2286 (0.16)
Europe (excluding Nordic)	113,989 (6.5)	82,(0.07)	205,(0.18)
Asia and Oceania	168,163 (9.6)	131 (0.08)	250 (0.15)
Africa	60,024 (3.4)	47 (0.08)	73 (0.12)
South America	18,407 (1.1)	29 (0.16)	30 (0.16)
Missing	4044 (0.2)	6	7
**Chronic disease pre-pregnancy** ^b^	175,375 (10.0)	230 (0.13)	447 (0.25)
Epilepsy	8730 (0.5)	2 (0.02)	20 (0.23)
Asthma	127,687 (7.3)	165 (0.13)	310 (0.24)
Kidney disease	8694 (0.5)	10 (0.12)	25 (0.29)
Systemic Lupus Erythematosus	1898 (0.1)	7 (0.37)	5 (0.26)
Inflammatory bowel disease	12,568 (0.7)	28 (0.22)	46 (0.37)
Hypertension	11,827 (0.7)	13 (0.11)	25 (0.21)
Diabetes	13,071 (0.7)	23 (0.18)	54 (0.41)
Gestational disease ^c^	96,978 (5.5)	166 (0.17)	289 (0.30)
Pre-eclampsia	52,206 (3.0)	102 (0.20)	195 (0.37)
Hypertension	19,912 (1.1)	31 (0.16)	51 (0.26)
Diabetes	19,234 (1.1)	29 (0.15)	43 (0.22)
Intrahepatic cholestasis of pregnancy (ICP)	8848 (0.5)	9 (0.10)	13 (0.15)
**Number of antenatal care visits**			
≤ 5	96,708 (5.8)	91 (0.09)	187 (0.19)
6–7	263,024 (15.7)	212 (0.08)	320 (0.12)
8–12	1,072,347 (63.8)	918 (0.09)	1645 (0.15)
≥ 13	248,498 (14.8)	343 (0.14)	566 (0.23)
Missing	74,292	73	133
**IVF conception**	50,483 (2.9)	70 (0.14)	125 (0.25)
**Previous history of caesarean section** ^d^			
Yes	164,401 (17.5)	299 (0.18)	406 (0.25)
No	772,757 (82.5)	358 (0.05)	591 (0.08)
Missing	40,729	20	40
**Gestational age** (weeks + days)			
≤ 36 + 6	96,107 (5.5)	130 (0.14)	242 (0.25)
37 + 0 to 41 + 6	1,535,345 (87.5)	1304 (0.08)	2325 (0.15)
≥ 42 + 0	122,621 (7.0)	198 (0.16)	280 (0.23)
Missing	796	5	4
**Multiple birth**			
Yes	25,794 (1.5)	46 (0.18)	85 (0.33)
No	1,728,770 (98.5)	1590 (0.09)	2763 (0.16)
Missing	305	1	3

IQR: Interquartile range; BMI: body mass index; SLE: systemic lupus erythematosus.

^a^Mother-infant dyads may not be unique due to the possibility of filed claims for multiple injuries.

^b^Chronic disease pre-pregnancy: indicated by any of the listed diseases (self-reported), with the possibility of women having multiple.

^c^Gestational disease (during pregnancy): indicated by any of the listed diseases (identified using ICD-10 codes), with the possibility of women having multiple.

^d^Previous history of caesarean section among multiparous women.

Several supplementary analyses were carried out to assess the robustness of estimates from the main analyses. Firstly, due to a high rate of missing for BMI (9.2%), the association between BMI and having a compensated claim was modelled (univariate and multivariable) using restricted cubic splines for, and categorically (with missing as a category) for analyses using the whole study population (Supplementary Figure S3). Secondly, we used generalised estimating equations to account for potential within-subject correlation between outcomes (clustered data) (Supplementary Table S3). Thirdly, missing data was managed in two ways: as a single category, or by complete case analyses. Finally, we modelled the outcome using multinomial logistic regression, with the following categories: having a compensated claim, rejected claim, or no claim (reference group) (Supplementary Table S4).

SAS (version 9.4; Cary, NC, USA) and R (version 4.1.2) were used for all data management and analyses. All methods were performed in accordance with the relevant guidelines and regulations.

### Ethical approval

This study was approved by the ethical review board in Stockholm (#2017-1308-31, granted 2017-08-16) who waived the need for written consent.

## Results

There was a higher proportion of compensated claims among women who were aged ≥ 35 years or had ≥ 13 years of education, compared to younger women and those with a lower education (Table 1). Additionally, a higher proportion of compensated claims were found among women who were nulliparous, born in Sweden or South America, delivered at week ≥ 42 + 0, and had certain chronic (systemic lupus erythematosus, inflammatory bowel disease, or diabetes) and gestational (pre-eclampsia, hypertension, diabetes, and intrahepatic cholestasis of pregnancy) diseases. While the number of filed maternal injury claims per year has more than doubled from 2000 to 2016, the number of compensated claims only increased slightly (Fig. 2a). From 2000 to 2005, the rate of filed claims remained relatively steady, while the rate of compensated claims, if at all, decreased marginally (Fig. 2b). On the other hand, from 2006 onwards, the rate of filed claims increased dramatically, while the rate of compensated claims increased only marginally.

Among the 1637 compensated maternal injury claims, very few resulted in moderate to severe disability or death (Fig. 1). By clinical classification, the largest number of compensated claims were due to ‘other’ injuries (24.5%) or ‘perineal tears/pelvic floor complications’ (21.8%), followed by surgical complications (11.5%) (Fig. 1). The large group of injuries classified as ‘other’ consists predominantly of medical errors, as well as diagnostic errors and heterogenous, unclassifiable injuries (Supplementary Table S1). The majority of these injuries resulted in sick leave, while 45% of women with ‘perineal tears/pelvic floor complications’ sustained a mild disability (Fig. 1).

In the univariate and multivariable exploratory analyses, a number of maternal characteristics were significantly associated with having a compensated maternal injury claim (Supplementary Table S2). Estimates for the multivariable analysis were similar when using only complete cases, and there was a suggestion that women aged ≥ 35 years (complete case OR 1.22, 95% CI 1.05, 1.42) and below-average in height (160–164 cm, OR 1.15, 95% CI 1.00, 1.31) had increased odds of having a compensated claim. Smoking at first antenatal visit and living with a partner were not associated with having a claim compensated.

For the final model, we used a top-down approach to identify the core set of maternal characteristics (from Supplementary Table S2) significantly associated with having a compensated claim. Compared to nulliparous women, those who had a prior caesarean section had increased odds of having a compensated claim (OR 1.32, 95% CI 1.14, 1.52), while women who had not had a caesarean section had reduced odds (OR 0.38, 95% CI 0.34, 0.44) (Fig. 3). There was some indication that women who were taller were less likely to have a compensated claim (OR 0.98, 95% CI 0.98, 0.99).

Compared to Nordic born women, there was some indication that women born in South America had increased odds of having a compensated maternal injury claim, although this was not statistically significant (OR 1.32: 95% CI 0.93, 2.07); while all other women born outside of the Nordic region had reduced odds of having a compensated claim (Fig. 3).

Women with a chronic (OR 1.31, 95% CI 1.13, 1.51) or gestational disease (OR 1.46, 95% CI 1.23, 1.74) had increased odds of having a compensated claim, compared to women without such diseases (Fig. 3). Additionally, women with more than the standard number of antenatal care visits (8–12) had increased odds of having a compensated claim (OR 1.40, 95% CI 1.23, 1.60).

Compared to women who gave birth at term (gestational week 37–39), women who gave birth at week 42 or later had significantly increased odds of having a compensated maternal injury claim (OR 1.73, 95% CI 1.47, 2.02) (Fig. 3). In the univariate analysis and multivariable analysis using all data, women with a preterm birth also had increased odds of having a compensated claim (Supplementary Table S2), although this was no longer significant in complete case analyses (OR 1.13: 95% CI 0.90, 1.40) (Fig. 3). Women with a multiple birth also had increased odds of having a compensated claim (OR 1.68, 95% CI 1.21, 2.33), compared to women delivering a singleton. Compared to women giving birth at the start of the study period (2000–2005), those giving birth later on had increased odds of having a compensated claim (2006–2011; OR 1.27, 95% CI 1.11, 1.45; 2012–2016; OR 1.58, 95% CI 1.38, 1.80).

Given that women can have repeated claims and pregnancies throughout the study period, we ran sensitivity analyses to account for possible clustering of data within subjects (Supplementary Table S3). Using Generalised Estimating Equations (GEE), we found the lowest quasi-information criteria for an independent covariance structure, indicating no evidence for within-subject correlation. When we further analysed the data with a multinomial outcome (Supplementary Table S4), the estimates using all data and complete cases was similar to the estimates shown in Supplementary Table S2.

## Discussion

In this large Swedish national cohort study of more than 1.75 million births between 2000 and 2016, we observed an almost two-fold increase in the total number of filed maternal injury claims; this increased dramatically from 2006 onwards and peaked in 2014. Despite this, the rate of compensated claims increased only marginally; of the 4488 filed maternal injury claims made, 36.5% were deemed as avoidable. Compared to maternal injury claims made at the start of our the study period (2000–2005), claims made between 2006 and 2011 and 2012 –2016, respectively, were 27% and 58% more likely to be compensated. Previously well known antenatal risk factors for adverse childbirth outcomes^14,21–24^ were also associated with having an compensated claim: previous caesarean section, post-term deliveries, region of birth, multiple pregnancy, chronic disease, gestational disease, and having > 13 antenatal visits.

The proportion of compensated claims in this study (36.5%) is modest compared to other studies estimating 50–74% of adverse obstetric events as avoidable^3–5^. We hypothesise this could be due to differences in the severity of adverse outcomes deemed as preventable, and some studies using clinicians and midwives internally to evaluate whether an outcome is adverse or preventable. Our data is from the National Patient Insurer (a professional external reviewer) who, after a standardised and thorough review of each claim, provides compensation only for preventable adverse events.

We found that women with a previous caesarean section had a 32% increased risk of having a compensated maternal injury claim. Previous studies have also found that having a previous caesarean section was associated with an increased risk of birth related asphyxia and uterine rupture^21,25^, and that the risk of adverse outcomes increases from gestational week 40 onwards^23,24^. Supporting this, we also found that women post 42 + 0 weeks of gestation had an increased risk of having a compensated maternal injury claim.

We further found that region of birth was associated with having filed a maternal injury claim: the proportion of filed claims was higher among women born in South America (0.32%), compared to the Nordic countries (0.26%), and lowest among women born in Africa (0.17%). While we found an increased risk of having a compensated maternal injury claim for women born in South America, this was not statistically significant, possibly due to a small numbers of observations. A previous study in 2013^26^ found that reported suboptimal care was a contributing factor of maternal deaths in Sweden among foreign born women; some of the factors related to delays or accessibility might also apply to our findings, including language and communication barriers leading to inadequate care and limited knowledge about how and when a complaint can be filed^26^. In addition to this, social support and lacking the skills to actually formulate a claim may make some women less likely to file a claim at all or have it compensated. Indeed, we found a higher rate of rejected claims among women with a lower education, smokers, with a BMI outside the ‘healthy weight’ range, and those not living with a partner.

Routine antenatal care visits are important for screening of risk factors associated with adverse pregnancy outcomes. In Sweden, primiparous women have on average 9 antenatal visits with a midwife, and multiparous women have slightly fewer (8.5)^27^. The increased risk of having a compensated maternal injury claim found among women who had > 13 antenatal visits was not surprising, given that a greater than average number of visits likely indicates complications during pregnancy, increased vulnerability and exposure to multiple interventions. Other factors we found associated with an increased risk of having a compensated maternal injury claim were also associated with a number of adverse outcomes, which likely increases the need for additional antenatal visits. Despite this, not all adverse outcomes will lead to a filed or compensated claim. Multiple pregnancy is associated with an increased risk of intrapartum hypoxia^28^, and we found a ~ 70% increased risk of having a compensated maternal injury claim among women with multiple gestation. Similarly, women with a chronic or gestational disease were more likely to have a compensated maternal injury claim.

Fortunately, very few of the claims resulted in moderate/severe disability or death. By clinical classification, ~ 22% of the compensated maternal injury claims were due to ‘perineal tears/pelvic floor complications’; an injury that can be minimised when good care and management is provided, including having two midwives present during the second stage of labor^29^. Mild disability was sustained for 45% of these women. While surgical complications were responsible for ~ 12% of the compensated maternal injury claims in our data set, the largest proportion (~ 25%) were due to injuries classified as ‘other’, the majority of which resulted in sick leave. This group of injuries consisted predominantly of medical errors, as well as diagnostic errors and heterogenous, unclassifiable injuries. A Swedish study^30^ across 21 hospitals estimates that, if all hospitals performed as well as the top 5 hospitals for each indicator, we could expect the following reductions in adverse outcomes: 23% for OASIS (obstetric anal sphincter injuries), 26% for postpartum haemorrhage, 30% for infections, and 42% for low 5-min Apgar score. This suggests that features of the healthcare system are important for mitigating the risk of complications during delivery.

Our study is strengthened with a large national sample size including over 1.7 million births and 4488 filed maternal injury claims, of which ~ 36% were compensated, allowing us to study compensated claims with high statistical power. Data was collected from high quality registers with prospectively collected data in standardized medical records from the antenatal, delivery, and postpartum periods.

As with all observational studies, there are limitations that need to be acknowledged. We were unable to review the medical records ourselves, and instead relied on the evaluation of the medical advisors at Löf, all experienced specialists in gynecology/obstetrics. Assessing whether an injury was avoidable or not, and thus eligible for compensation, was based on the woman’s own report and medical files from the delivery hospital. Therefore, there could be variation in the evaluation of individual cases by Löf, depending on the reviewer responsible for judging the evidence; however, each claim is often evaluated by two medical advisors. Continuous internal quality assessments conducted by Löf (which are not published externally) indicate that any such variation is very small, both with regard to individual claims and also over time.

Compensated claims were a proxy for receiving suboptimal care during childbirth, since compensation was only given for maternal injuries evaluated as avoidable, and not merely based on the outcome; for example, having a severe perineal tear was not grounds for compensation unless evidence suggested that the injury was avoidable. Additionally, there may be women who suffered an avoidable injury but did not file a maternal injury claim, leading to outcome misclassification. Hence, some women included in our reference group may have indeed suffered an avoidable injury, although our results would then rather underestimate the true association. We acknowledge that a number of factors during childbirth will also influence the risk of maternal injury. This study was conducted as an important initial investigation of trends and predictive risk factors (upon entering the delivery ward) for having an avoidable maternal injury, as measured by compensated claims. Although complex, there is a need for in-depth investigation of factors during the labour and birth that contribute to the risk of having an avoidable maternal injury. Such research is crucial for gaining insight into how the quality of care can be enhanced to improve maternal outcomes.

To minimise the risk of misclassification when subclassifying injuries (completed by the first author), complicated cases were discussed with co-authors—senior obstetrician, OS, and Löf representative, PG—until a consensus was reached. Additionally, while we adjusted for confounders in our multivariable regression models, unknown and residual confounding may still be present.

In conclusion, perineal and pelvic floor injuries, as well as medical and diagnostic errors, were responsible for the majority of compensated maternal injury claims. Many of the risk factors for having a compensated maternal injury claim (a proxy for having an avoidable injury) are also known risks for having an adverse outcome during delivery. We found that women with a previous caesarean section, post term delivery, a chronic disease or gestational disease, > 13 antenatal visits, multiple pregnancy had an increased risk of having a compensated maternal injury claim. Knowledge of these risk factors can therefore increase awareness among clinicians/midwives about subgroups of patients who may require earlier initiation of preventative measures to avoid adverse outcomes. Further research is needed to understand why the number of filed claims has been increasing and specific ways in which health professionals and maternity wards can improve the quality and organisation of care to mitigate the risk of avoidable injuries during childbirth.

### Supplementary Information

Supplementary Information.

## Data Availability

The data that supports the findings of this study are available from Löf (maternal injury claims), which were then linked to Swedish Register data (available from Socialstyrelsen and Statistics Sweden) using the Swedish Personal Idenification Number. Applications to use this data must be made to the respective bodies. Further information about accessing this data can be made to the corresponding author.
