# Supplementary material for: Maternal and pregnancy predictive risk factors for having a compensated maternal injury claim: a Swedish nationwide cohort study
Source: Sci Rep. 2023 Dec 8;13:21731. doi: 10.1038/s41598-023-49234-7 (PMC10709443; doi:10.1038/s41598-023-49234-7)
Supplement: Supplementary file 1 — Supplementary Information. [file 41598_2023_49234_MOESM1_ESM.docx]

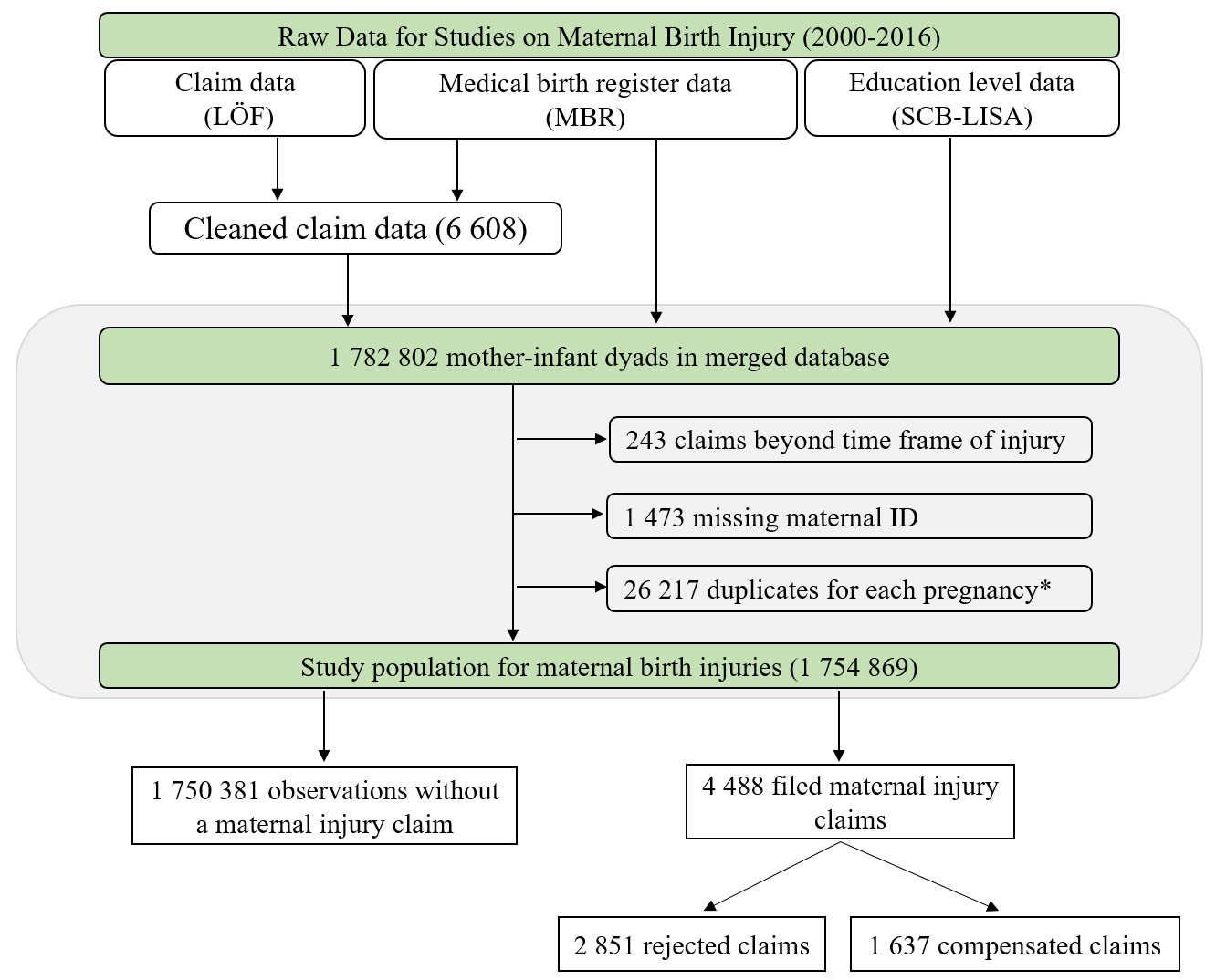


**Supplementary Figure S1.** Flowchart of the study population, including mother-infant dyads without a maternal injury claim, as well as those with a rejected or compensated claim.

* Duplicate rows for the same claim per pregnancy are removed. Multiple rows are included for women with multiple unique claims per pregnancy

*LÖF*: The regions mutual insurance company for patient injuries.
*MBR*: Medical birth register.
*SCB-LISA*: Statistics Sweden - Longitudinal Integrated Database for Health Insurance and Labour Market Studies.


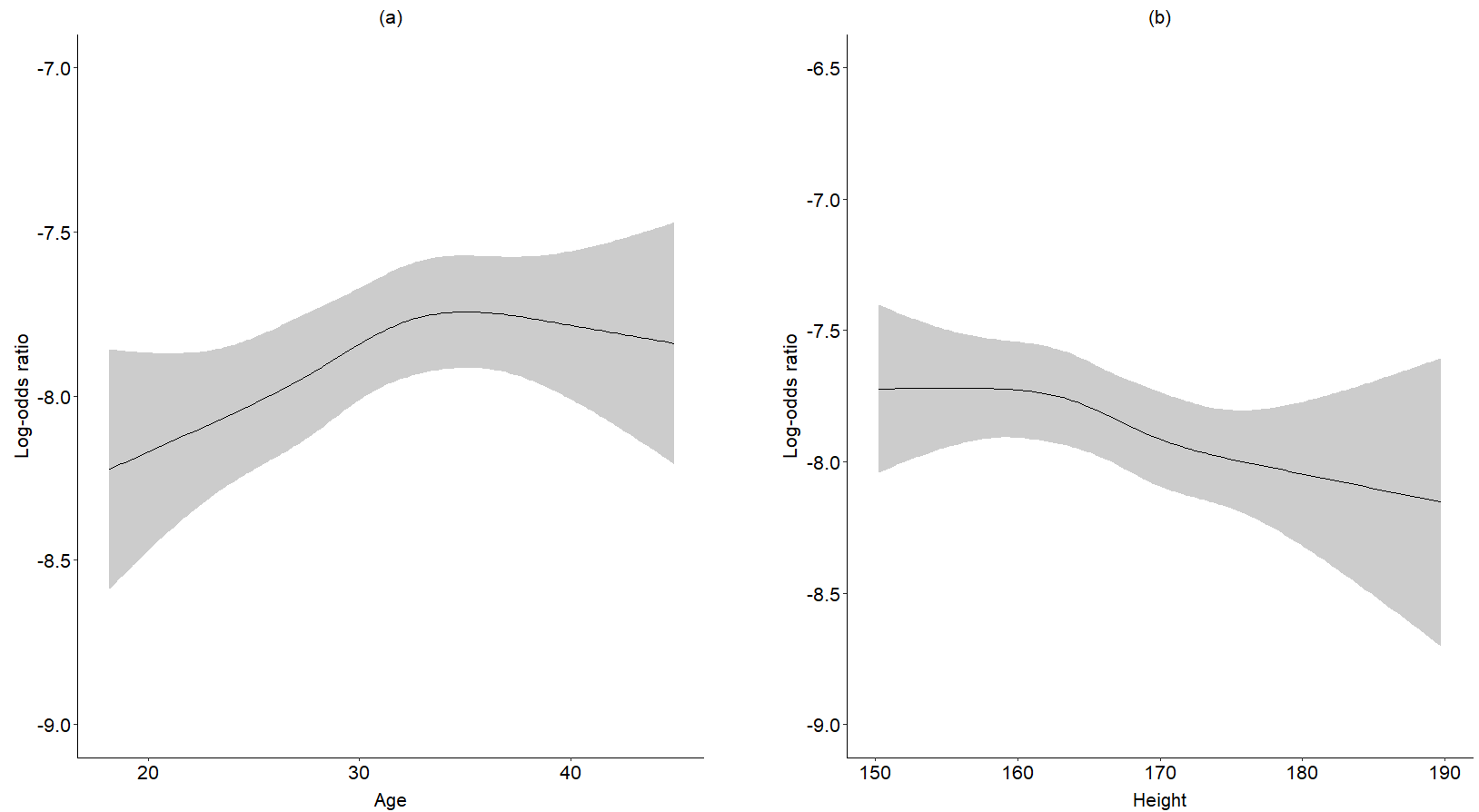


**Supplementary Figure S2.** Restricted cubic splines (with three degrees of freedom) to explore the possible non-linear effect of a) age and b) height on having a compensated maternal injury claim, modelled using multivariable logistic regression including calendar year and all characteristics in Table 1 (excluding BMI)


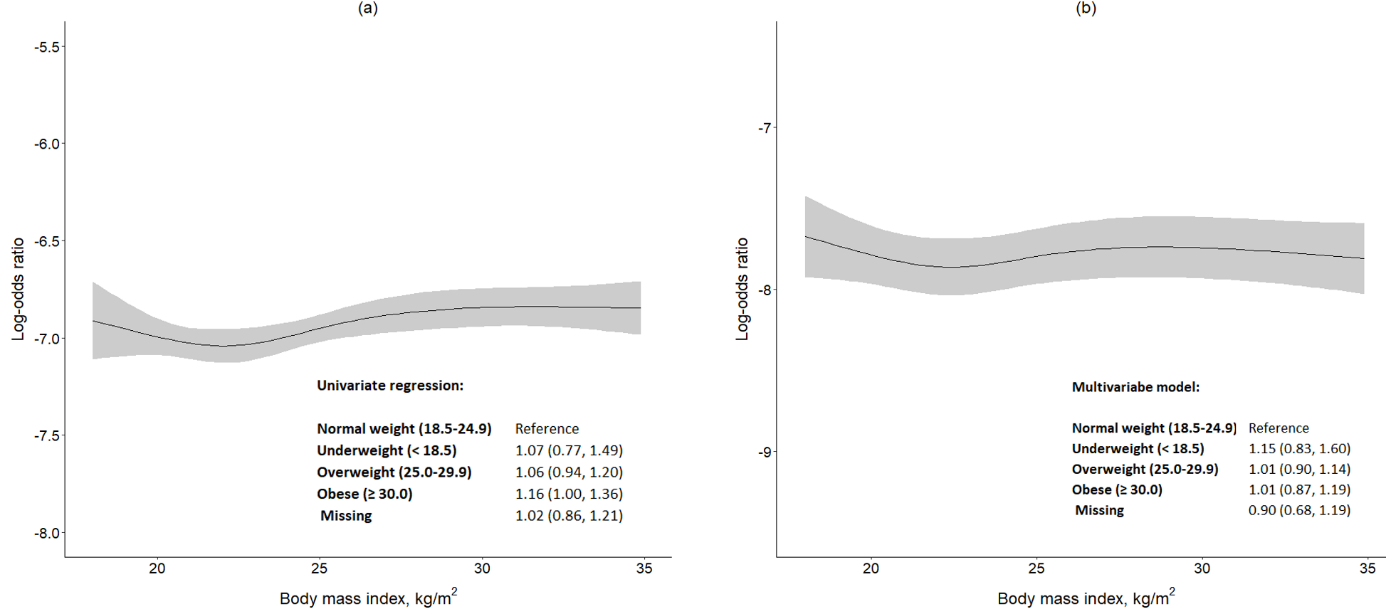


**Supplementary Figure S3.** Supplementary analyses exploring the association between body mass index (BMI) and having a compensated claim, (due to a large proportion of missing BMI data - 9.2%), modelled using (a) univariate regression and b) multivariable regression, including maternal and pregnancy characteristics (listed in the Methods section).

Log-odds ratio (95% confidence intervals (CI) presented when modelling BMI as a continuous variable (using restricted cubic splines among complete cases) and odds ratio (95% CI) when modelling BMI as a categorical variable (all data, including missing as a category)

**Supplementary Table S1.** Subclassification of compensated maternal injury claims (a proxy for sub-optimal care) classified as ‘other’ (n=401, 25.7% of all compensated claims).

| **Type of injury/complication** | **n** | **%** |
| --- | --- | --- |
| Medical | 120 | 29.9 |
| Unclassifiable | 79 | 19.7 |
| Diagnostic | 60 | 14.9 |
| Pain/hypoesthesia | 27 | 6.7 |
| Coagulation/ thrombosis | 22 | 5.5 |
| Orthopedic/ joints | 19 | 4.7 |
| Surgical | 14 | 3.5 |
| Gynecological | 8 | 2.0 |
| Asphyxia | 7 | 1.75 |
| Abdominal | 7 | 1.75 |
| Brain | 6 | 1.5 |
| Preeclampsia/ HELLP/ Eclampsia | 6 | 1.5 |
| Deterioration of underlying condition | 5 | 1.3 |
| Skin | 5 | 1.3 |
| Cardiac | 4 | 1.0 |
| Respiratory | 4 | 1.0 |
| Foreign body | 4 | 1.0 |
| Eye | 2 | 0.5 |
| Infectious diseases | 1 | 0.3 |
| Kidney | 1 | 0.3 |

**Supplementary Table S2.** The association between maternal and pregnancy characteristics and having a compensated maternal injury claim (a proxy for sub-optimal care). Odds ratios (95% C.I.) from univariate and multivariable logistic regression models (N=1 754 869).

| **Characteristics** | **Having a compensated maternal injury claim**  **Odds ratio (95% CI)** | | |
| --- | --- | --- | --- |
|  | **Univariate analysis** | **Multivariable analysis ^0^** | |
|  | **all data ^1^** | **all data ^1^** | **complete cases ^2^** |
| Maternal age (years) |  |  |  |
| ≤ 24 | 0.92 (0.78, 1.09) | 0.84 (0.71, 1.00) | 0.87 (0.73, 1.04) |
| 25-29 | 1.00 | 1.00 | 1.00 |
| 30-34 | 1.06 (0.94, 1.20) | 1.15 (1.01, 1.30) | 1.11 (0.98, 1.27) |
| ≥ 35 | 1.18 (1.03, 1.35) | 1.28 (1.11, 1.48) | 1.22 (1.05, 1.42) |
|  |  |  |  |
| Parity and previous history of caesarean section |  |  |  |
| Nulliparous | 1.00 | 1.00 | 1.00 |
| Parous without previous caesarean | 0.37 (0.33, 0.42) | 0.37 (0.33, 0.43) | 0.38 (0.33, 0.44) |
| Parous with previous caesarean | 1.47 (1.29, 1.68) | 1.34 (1.16, 1.53) | 1.35 (1.16, 1.56) |
|  |  |  |  |
| Maternal height (cm) |  |  |  |
| ≤ 159 | 1.04 (0.89, 1.23) | 1.05 (0.89, 1.25) | 1.05 (0.88, 1.26) |
| 160-164 | 1.13 (0.99, 1.29) | 1.14 (1.00, 1.30) | 1.15 (1.00, 1.31) |
| 165-169 | 1.00 | 1.00 | 1.00 |
| ≥ 170 | 0.91 (0.79, 1.04) | 0.89 (0.78, 1.02) | 0.90 (0.78, 1.02) |
|  |  |  |  |
| Smoking at first antenatal visit | 0.98 (0.80, 1.19) | 1.11 (0.90, 1.35) | 1.14 (0.92, 1.39) |
|  |  |  |  |
| Years of formal education |  |  |  |
| ≤ 9 | 0.78 (0.65, 0.92) | 0.95 (0.78, 1.15) | 0.91 (0.73, 1.11) |
| 10-12 | 0.90 (0.81, 1.00) | 0.99 (0.89, 1.11) | 0.97 (0.86, 1.09) |
| ≥ 13 | 1.00 | 1.00 | 1.00 |
|  |  |  |  |
| Living with partner | 0.98 (0.80, 1.22) | 0.91 (0.73, 1.13) | 0.90 (0.71, 1.13) |
|  |  |  |  |
| Region of birth (mother) |  |  |  |
| Nordic | 1.00 | 1.00 | 1.00 |
| Europe (excluding Nordic) | 0.75 (0.59, 0.93) | 0.76 (0.60, 0.94) | 0.79 (0.61, 0.99) |
| Asia and Oceania | 0.81 (0.67, 0.97) | 0.82 (0.67, 0.99) | 0.83 (0.67, 1.02) |
| Africa | 0.81 (0.61, 1.09) | 0.81 (0.59, 1.08) | 0.80 (0.55, 1.12) |
| South America | 1.63 (1.13, 2.36) | 1.52 (1.02, 2.16) | 1.49 (0.96, 2.18) |
|  |  |  |  |
| Chronic disease ^b^ | 1.47 (1.28, 1.69) | 1.31 (1.13, 1.50) | 1.27 (1.09, 1.47) |
| Gestational disease ^c^ | 1.93 (1.64, 2.27) | 1.47 (1.24, 1.73) | 1.49 (1.24, 1.77) |
|  |  |  |  |
| Number of antenatal care visits |  |  |  |
| ≤ 5 | 1.10 (0.89, 1.36) | 1.00 (0.79, 1.24) | 1.00 (0.77, 1.26) |
| 6-7 | 0.94 (0.81, 1.09) | 1.00 (0.86, 1.17) | 0.96 (0.82, 1.13) |
| 8-12 | 1.00 | 1.00 | 1.00 |
| ≥ 13 | 1.61 (1.42, 1.83) | 1.40 (1.23, 1.59) | 1.41 (1.23, 1.60) |
|  |  |  |  |
| IVF conception | 1.51 (1.18, 1.92) | 1.04 (0.81, 1.33) | 1.05 (0.81, 1.35) |
|  |  |  |  |
| Gestational age (weeks + days) |  |  |  |
| ≤ 36 + 6 | 1.59 (1.33, 1.91) | 1.26 (1.03, 1.53) | 1.12 (0.89, 1.40) |
| 37+0 to 41+6 | 1.00 | 1.00 | 1.00 |
| ≥ 42+0 | 1.90 (1.64, 2.21) | 1.74 (1.49, 2.02) | 1.71 (1.45, 2.00) |
|  |  |  |  |
| Multiple birth | 1.94 (1.45, 2.60) | 1.61 (1.17, 2.17) | 1.67 (1.18, 2.30) |
|  |  |  |  |
| Calendar period |  |  |  |
| 2000 - 2005 | 1.00 | 1.00 | 1.00 |
| 2006 - 2011 | 1.22 (1.07, 1.38) | 1.25 (1.10, 1.42) | 1.26 (1.10, 1.44) |
| 2012 - 2016 | 1.49 (1.32, 1.69) | 1.57 (1.38, 1.78) | 1.57 (1.37, 1.80) |
|  |  |  |  |

CI: confidence interval; BMI: body mass index.

^0^ Multivariable regression includes the following variables: maternal age, parity and previous history of caesarean section, maternal height, smoking status at first antenatal visit, years of formal education, living with partner, region of birth (mother), chronic disease (defined as any of the following: epilepsy, asthma, kidney disease, systemic lupus erythematosus, inflammatory bowel disease (ulcerative colitis or Crohn’s), chronic hypertension, or chronic diabetes), gestational disease (defined as any of the following: preeclampsia, gestational hypertension, diabetes, or intrahepatic cholestasis of pregnancy (ICP)), number of antenatal care visits, in vitro fertilization conception, gestational age, multiple births, and calendar year.

^1^ Missing data as a category

^2^ Missing data excluded

**Supplementary Table S3.** The association between maternal and pregnancy characteristics and having a compensated maternal injury claim (a proxy for sub-optimal care), accounting for a potential correlation of outcomes within same subjects using GEE approaches (and including missing as a category). Odds ratios (95% CI) from univariate and multivariable logistic regression models (N=1 754 869).

| **Characteristics** | **Having a compensated maternal injury claim**  **Odds ratio (95% CI)** | | |
| --- | --- | --- | --- |
|  | **GEE-independent** | **GEE-exchangeable** | **GEE-unstructured** |
| Maternal age (years) |  |  |  |
| ≤ 24 | 0.84 (0.71, 1.00) | 0.85 (0.72, 1.01) | 0.85 (0.72, 1.01) |
| 25-29 | 1.00 | 1.00 | 1.00 |
| 30-34 | 1.15 (1.01, 1.30) | 1.13 (1.00, 1.29) | 1.13 (1.00, 1.29) |
| ≥ 35 | 1.28 (1.11, 1.48) | 1.26 (1.09, 1.46) | 1.26 (1.09, 1.46) |
|  |  |  |  |
| Parity and previous history of caesarean section |  |  |  |
| Nulliparous | 1.00 | 1.00 | 1.00 |
| Parous without previous caesarean | 0.37 (0.33, 0.43) | 0.37 (0.32, 0.42) | 0.37 (0.32, 0.42) |
| Parous with previous caesarean | 1.34 (1.16, 1.54) | 1.30 (1.12, 1.50) | 1.30 (1.12, 1.50) |
|  |  |  |  |
| Maternal height (cm) |  |  |  |
| ≤ 159 | 1.05 (0.89, 1.25) | 1.04 (0.88, 1.23) | 1.04 (0.88, 1.23) |
| 160-164 | 1.14 (1.00, 1.30) | 1.12 (0.98, 1.28) | 1.12 (0.98, 1.28) |
| 165-169 | 1.00 | 1.00 | 1.00 |
| ≥ 170 | 0.89 (0.78, 1.02) | 0.88 (0.77, 1.00) | 0.88 (0.77, 1.00) |
|  |  |  |  |
| Smoking at first antenatal visit |  |  |  |
| Smokers | 1.11 (0.90, 1.36) | 1.12 (0.91, 1.38) | 1.12 (0.91, 1.38) |
| Non-smoker | 1.00 | 1.00 | 1.00 |
|  |  |  |  |
| Years of formal education |  |  |  |
| ≤ 9 | 0.95 (0.77, 1.16) | 0.94 (0.77, 1.15) | 0.94 (0.77, 1.15) |
| 10-12 | 0.99 (0.89, 1.11) | 0.99 (0.88, 1.10) | 0.99 (0.88, 1.10) |
| ≥ 13 | 1.00 | 1.00 | 1.00 |
|  |  |  |  |
| Living with partner |  |  |  |
| Yes | 1.00 | 1.00 | 1.00 |
| No | 0.91 (0.73, 1.14) | 0.92 (0.73, 1.16) | 0.92 (0.73, 1.16) |
|  |  |  |  |
| Region of birth (mother) |  |  |  |
| Nordic | 1.00 | 1.00 | 1.00 |
| Europe (excluding Nordic) | 0.76 (0.60, 0.95) | 0.73 (0.58, 0.92) | 0.73 (0.58, 0.92) |
| Asia and Oceania | 0.82 (0.67, 0.99) | 0.83 (0.68, 1.01) | 0.83 (0.68, 1.01) |
| Africa | 0.81 (0.59, 1.11) | 0.83 (0.61, 1.14) | 0.83 (0.61, 1.14) |
| South America | 1.52 (1.04, 2.20) | 1.56 (1.08, 2.27) | 1.56 (1.08, 2.27) |
|  |  |  |  |
| Chronic Disease ^b^ |  |  |  |
| No | 1.00 | 1.00 | 1.00 |
| Yes | 1.31 (1.13, 1.51) | 1.30 (1.13, 1.50) | 1.30 (1.13, 1.50) |
|  |  |  |  |
| Gestational Disease ^c^ |  |  |  |
| No | 1.00 | 1.00 | 1.00 |
| Yes | 1.47 (1.24, 1.74) | 1.46 (1.23, 1.74) | 1.46 (1.23, 1.74) |
|  |  |  |  |
| Number of antenatal care visits |  |  |  |
| ≤ 5 | 1.00 (0.80, 1.25) | 1.03 (0.83, 1.29) | 1.03 (0.83, 1.29) |
| 6-7 | 1.00 (0.86, 1.17) | 1.02 (0.88, 1.20) | 1.02 (0.88, 1.20) |
| 8-12 | 1.00 | 1.00 | 1.00 |
| ≥ 13 | 1.40 (1.23, 1.60) | 1.40 (1.23, 1.60) | 1.40 (1.23, 1.60) |
|  |  |  |  |
| IVF conception |  |  |  |
| No | 1.00 | 1.00 | 1.00 |
| Yes | 1.04 (0.81, .35) | 1.05 (0.81, 1.35) | 1.05 (0.81, 1.35) |
|  |  |  |  |
| Gestational age (weeks + days) |  |  |  |
| ≤ 36 + 6 | 1.26 (1.03, 1.54) | 1.24 (1.01, 1.53) | 1.24 (1.01, 1.53) |
| 37+0 to 41+6 | 1.00 | 1.00 | 1.00 |
| ≥ 42+0 | 1.74 (1.49, 2.03) | 1.73 (1.48, 2.02) | 1.73 (1.48, 2.02) |
|  |  |  |  |
| Multiple births |  |  |  |
| No | 1.00 | 1.00 | 1.00 |
| Yes | 1.61 (1.18, 2.21) | 1.67 (1.22, 2.28) | 1.67 (1.22, 2.28) |
|  |  |  |  |
| Calendar period |  |  |  |
| 2000 - 2005 | 1.00 | 1.00 | 1.00 |
| 2006 - 2011 | 1.25 (1.10, 1.42) | 1.25 (1.10, 1.42) | 1.25 (1.10, 1.42) |
| 2012 - 2016 | 1.57 (1.38, 1.78) | 1.56 (1.37, 1.78) | 1.56 (1.37, 1.78) |
|  |  |  |  |
| Quasi information criterion | 25441.64 | 25444.18 | 25444.18 |

GEE: generalized estimating equations; CI: confidence interval; BMI: body mass index

^1^ Multivariable regression includes the following variables: maternal age, parity and previous history of caesarean section, maternal height, smoking status at first antenatal visit, years of formal education, living with partner, region of birth (mother), chronic disease (defined as any of the following: epilepsy, asthma, kidney disease, systemic lupus erythematosus, inflammatory bowel disease (ulcerative colitis or Crohn’s), chronic hypertension, or chronic diabetes), gestational disease (defined as any of the following: preeclampsia, gestational hypertension, diabetes, or intrahepatic cholestasis of pregnancy (ICP)), number of antenatal care visits, in vitro fertilization conception, gestational age, multiple births, and calendar year

**Supplementary Table S4.** The association between maternal and pregnancy characteristics and having a rejected or compensated maternal injury claim, with *no any claim* as the reference. Odds ratios (95% CI) from multinomial logistic regression models ^0^ (N=1 754 869).

| **Characteristics** | **Having a maternal injury claim**  **Odds ratio ^1^ (95% CI)** | | | |
| --- | --- | --- | --- | --- |
|  | **All data ^2^** | | **Complete cases ^3^** | |
|  | **Rejected** | **Compensated** | **Rejected** | **Compensated** |
| Maternal age (years) |  |  |  |  |
| ≤ 24 | 0.99 (0.89, 1.11) | 0.86 (0.72, 1.02) | 0.99 (0.87, 1.12) | 0.87 (0.73, 1.05) |
| 25-29 | 1.00 | 1.00 | 1.00 | 1.00 |
| 30-34 | 1.00 (0.91, 1.10) | 1.09 (0.96, 1.24) | 1.01 (0.91, 1.11) | 1.11 (0.98, 1.27) |
| ≥ 35 | 0.99 (0.88, 1.10) | 1.18 (1.02, 1.36) | 1.04 (0.92, 1.17) | 1.22 (1.05, 1.42) |
|  |  |  |  |  |
| Parity and previous history of caesarean section |  |  |  |  |
| Nulliparous | 1.00 | 1.00 | 1.00 | 1.00 |
| Parous without previous caesarean | 0.36 (0.33, 0.40) | 0.38 (0.33, 0.43) | 0.36 (0.32, 0.39) | 0.38 (0.33, 0.44) |
| Parous with previous caesarean | 1.05 (0.94, 1.17) | 1.30 (1.13, 1.50) | 1.04 (0.92, 1.17) | 1.35 (1.16, 1.57) |
|  |  |  |  |  |
| Maternal height (cm) |  |  |  |  |
| ≤ 159 | 1.16 (1.03, 1.31) | 1.16 (0.98, 1.38) | 1.18 (1.04, 1.34) | 1.05 (0.88, 1.26) |
| 160-164 | 1.02 (0.92, 1.13) | 1.18 (1.03, 1.36) | 1.00 (0.90, 1.11) | 1.15 (1.00, 1.31) |
| 165-169 | 1.00 | 1.00 | 1.00 | 1.00 |
| ≥ 170 | 0.93 (0.84, 1.02) | 0.98 (0.86, 1.12) | 0.92 (0.83, 1.02) | 0.89 (0.78, 1.02) |
|  |  |  |  |  |
| Smoking at first antenatal visit | 1.10 (0.95, 1.28) | 1.17 (0.96, 1.43) | 1.21 (1.04, 1.41) | 1.14 (0.92, 1.40) |
|  |  |  |  |  |
| Years of formal education |  |  |  |  |
| ≤ 9 | 0.98 (0.85, 1.13) | 0.97 (0.80, 1.18) | 0.98 (0.84, 1.14) | 0.91 (0.74, 1.12) |
| 10-12 | 1.14 (1.05, 1.24) | 0.98 (0.88, 1.10) | 1.14 (1.04, 1.24) | 0.97 (0.86, 1.09) |
| ≥ 13 | 1.00 | 1.00 | 1.00 | 1.00 |
|  |  |  |  |  |
| Living with partner | 1.03 (0.88, 1.19) | 0.96 (0.77, 1.19) | 1.06 (0.90, 1.24) | 0.90 (0.72, 1.14) |
|  |  |  |  |  |
| Region of birth (mother) |  |  |  |  |
| Nordic | 1.00 | 1.00 | 1.00 | 1.00 |
| Europe (excluding Nordic) | 1.16 (1.01, 1.33) | 0.78 (0.62, 0.98) | 1.12 (0.96, 1.31) | 0.79 (0.62, 1.00) |
| Asia and Oceania | 0.90 (0.78, 1.03) | 0.81 (0.66, 0.98) | 0.88 (0.76, 1.02) | 0.83 (0.67, 1.02) |
| Africa | 0.79 (0.62, 0.99) | 0.89 (0.66, 1.20) | 0.69 (0.52, 0.91) | 0.80 (0.56, 1.14) |
| South America | 0.79 (0.53, 1.16) | 0.93 (0.57, 1.50) | 0.91 (0.62, 1.35) | 1.48 (0.99, 2.24) |
|  |  |  |  |  |
| Chronic Disease ^b^ | 1.48 (1.34, 1.64) | 1.30 (1.12, 1.50) | 1.49 (1.34, 1.65) | 1.27 (1.10, 1.48) |
| Gestational Disease ^c^ | 1.37 (1.21, 1.56) | 1.33 (1.12, 1.59) | 1.44 (1.26, 1.64) | 1.49 (1.25, 1.78) |
|  |  |  |  |  |
| Number of antenatal care visits |  |  |  |  |
| ≤ 5 | 1.08 (0.93, 1.27) | 1.11 (0.89, 1.39) | 1.03 (0.87, 1.22) | 1.00 (0.78, 1.27) |
| 6-7 | 0.80 (0.71, 0.90) | 0.98 (0.84, 1.15) | 0.82 (0.73, 0.93) | 0.96 (0.82, 1.13) |
| 8-12 | 1.00 | 1.00 | 1.00 | 1.00 |
| ≥ 13 | 1.40 (1.27, 1.55) | 1.47 (1.29, 1.67) | 1.40 (1.27, 1.55) | 1.41 (1.23, 1.61) |
|  |  |  |  |  |
| IVF conception | 1.03 (0.85, 1.24) | 1.23 (0.97, 1.57) | 1.05 (0.87, 1.28) | 1.05 (0.82, 1.36) |
|  |  |  |  |  |
| Gestational Age, weeks + days |  |  |  |  |
| ≤ 36 + 6 | 1.32 (1.14, 1.52) | 1.14 (0.92, 1.40) | 1.26 (1.08, 1.49) | 1.12 (0.90, 1.41) |
| 37+0 to 41+6 | 1.00 | 1.00 | 1.00 | 1.00 |
| ≥ 42+0 | 1.35 (1.19, 1.52) | 1.92 (1.66, 2.23) | 1.37 (1.20, 1.56) | 1.71 (1.46, 2.01) |
|  |  |  |  |  |
| Multiple births | 1.70 (1.36, 2.13) | 1.42 (1.01, 1.99) | 1.74 (1.36, 2.22) | 1.67 (1.20, 2.34) |
|  |  |  |  |  |
| Calendar period |  |  |  |  |
| 2000 - 2005 | 1.00 | 1.00 | 1.00 | 1.00 |
| 2006 - 2011 | 2.33 (2.09, 2.61) | 1.40 (1.23, 1.60) | 2.28 (2.02, 2.57) | 1.26 (1.10, 1.44) |
| 2012 - 2016 | 3.51 (3.15, 3.92) | 1.65 (1.44, 1.88) | 3.35 (2.98, 3.77) | 1.57 (1.37, 1.80) |
|  |  |  |  |  |

CI: confidence interval; BMI: body mass index.

^0^ The regression model includes the following variables: maternal age, parity and previous history of caesarean section, maternal height, smoking status at first antenatal visit, years of formal education, living with partner, region of birth (mother), chronic disease (defined as any of the following: epilepsy, asthma, kidney disease, systemic lupus erythematosus, inflammatory bowel disease (ulcerative colitis or Crohn’s), chronic hypertension, or chronic diabetes), gestational disease (defined as any of the following: preeclampsia, gestational hypertension, diabetes, or intrahepatic cholestasis of pregnancy (ICP)), number of antenatal care visits, in vitro fertilization conception, gestational age, multiple births, and calendar year.

^1^ Within the literature, *relative risk ratio* is also interpreted as the association measure from multinomial logistic regression
^2^ Missing data as a category

^3^ Missing data excluded
